# Waning neutralizing antibodies through 180 days after homologous and heterologous boosters of inactivated COVID-19 vaccine

**DOI:** 10.3389/fpubh.2025.1478627

**Published:** 2025-01-28

**Authors:** Zhifei Chen, Fangqin Xie, Hairong Zhang, Dong Li, Suhan Zhang, Mengping Zhang, Junrong Li, Jianfeng Xie, Lina Zhang, Xiuhui Yang, Dongjuan Zhang

**Affiliations:** ^1^Fujian Provincial Center for Disease Control and Prevention, Fuzhou, China; ^2^Zhangping Center for Disease Control and Prevention, Zhangping, China

**Keywords:** COVID-19, SARS-CoV-2, inactivated vaccine, heterologous immunization, homologous immunization, neutralizing antibodies

## Abstract

To enhance the personal immunity to COVID-19, a third booster dose of inactivated COVID-19 vaccines program campaign was implemented in China. Our study endeavored to compare the dynamics of neutralizing antibodies generated by four distinct booster vaccines against three kinds of live SARS-CoV-2 virus (wild-type, Delta AY.23, and Omicron BA5.2). This cohort study involved 320 healthy individuals, who were randomly assigned to four groups, to receive boosters with inactivated vaccine (COVac and BIBP), the adenovirus type-5-vectored vaccine (Convidecia), and the recombinant protein-based vaccine (Zifivax), respectively, all the vaccines studied had the Wuhan variant as their parental variant. Participants were recruited from December 2021 to June 2022, with a follow-up period of 180 days. We evaluated humoral immune responses and their longevity by measuring the geometric mean titers (GMTs) of neutralizing antibodies against the SARS-CoV-2 virus at various time points post-boost. After 180 days of follow-up, 310 participants completed the study. Across all booster groups, neutralizing antibodies against the wild-type virus declined sharply within the first 90 days, accounting for an 81.24 to 92.34% reduction, then slowed down with gradually decreasing decay rates. By day 14 of post-boost, the ability to neutralize the Delta variant slightly diminished compared to the wild-type, whereas neutralizing antibodies against the Omicron variant exhibited a more pronounced decline, ranging from 10.78 to 19.88 times lower than those against the wild-type. Notably, heterologous boosting with the Convidecia vaccine maintained higher GMTs of neutralizing antibodies against both Delta and Omicron variants compared to the other boosters. At 180 days of post-boost, GMTs of neutralizing antibodies against SARS-CoV-2 had substantially decreased, yet individuals who received the Convidecia vaccine still exhibited higher titers than those who received other boosters. In summary, neutralizing antibody levels significantly waned 180 days after the third vaccine dose, with the most pronounced decline occurring within the initial 90 days. Heterologous boosting with Convidecia demonstrated a more robust, durable, and broad humoral immune response compared to boosting with inactivated vaccines or Zifivax, suggesting that adenovirus vector vaccines possess a special advantage in the realm of vaccine development for preventing infectious diseases.

## Introduction

The coronavirus disease 2019 (COVID-19), caused by severe acute respiratory syndrome coronavirus 2 (SARS-CoV-2), has severely impacted the health, economy, and society all over the world. As of 14 January 2024, COVID-19 has caused 774 million illnesses and over 7.0 million deaths (1). Mass vaccination campaigns are an important means to reduce the burden of disease and the subsequent economic recovery. A total of 13.59 billion doses have been administered globally, with 3.52 billion doses completed in China (1). More than 85% of China’s population has received primary immunization, and 57% have been vaccinated with at least one booster dose (1). The inactivated vaccines CoronaVac and BBIBP-CorV were the first home-grown vaccines to be used, playing a major role in primary immunization. These were followed by the adenovirus type-5-vectored vaccine Convidecia and the recombinant Novel coronavirus vaccine (CHO cells), Zifivax. The first three were included in the list of emergency use by the WHO (2). As the epidemic improves, more COVID-19 vaccines have been authorized for use against the disease.

While the antibody responses elicited by the inactivated vaccine (BBIBP-CorV) were lower when compared to the mRNA-based vaccine (BNT162b2) and the protein subunit vaccine (Covovax) (3), it induced a comprehensive immune response by exposing the complete virus, demonstrating significant immunogenicity and protective efficacy (4–7). Nevertheless, irrespective of the vaccine platform utilized, reports indicated that neutralizing antibody responses declined by 6 months following the initial immunization (8–10). As the waning of neutralizing antibodies against SARS-CoV-2 coincides with the emergence of the new variants, the effectiveness of COVID-19 vaccines has declined over time (11–13), necessitating booster vaccinations. In November 2021, China launched the booster immunization of the COVID-19 vaccine, boosting the third dosage for 18-year-olds and above who had completed the primary immunization. The preliminary results of our study, which began in December 2021, found that the neutralizing antibody in individuals who had been boosted increased significantly 14 days later, but began to decline at 28 days (14). With the continuous emergence of SARS-CoV-2 variants, many variants have shown increased transmission capabilities and enhanced ability to evade neutralizing antibodies, posing serious challenges to the protective effect of vaccines. In this study, we analyzed the neutralizing antibody responses and dynamic characteristics of individuals 180 days after the primary boost regimen, aiming to provide essential data for evaluating the effectiveness of COVID-19 vaccines and informing the adjustment of immunization strategies.

## Materials and methods

### Study population

**Figure 1:**
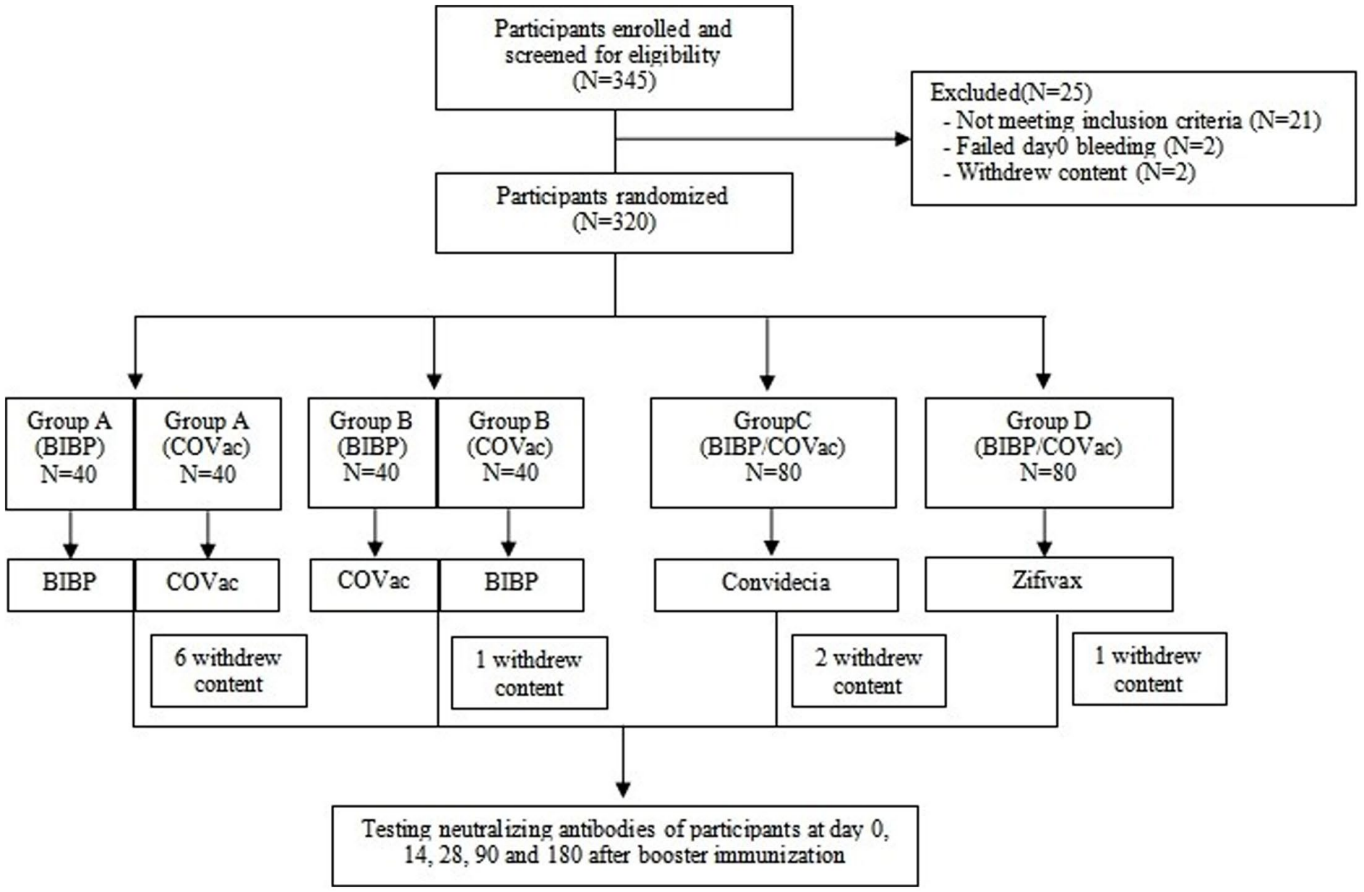
Recruitment of participants, follow-up, and testing. This study involved a prospective cohort of healthy individuals who were vaccinated at least 6 months with two doses of inactivated vaccine. During the study period, participants were followed 180 days after receipt of the third dose. BIBP: The abbreviation of Sinopharm BIBP (inactivated vaccine), COVac: the abbreviation of CoronaVac (inactivated vaccine), Convidecia: the abbreviation of Convidecia adenovirus type-5-vectored COVID-19 vaccine (adenovirus vector vaccine), and Zifivax: the abbreviation of Zifivax recombinant novel coronavirus vaccine (CHO cells) (recombinant protein-based vaccine).

This cohort study was carried out at Zhangping City Center for Disease Control and Prevention located in Longyan, Fujian (14). Enrollment of participants occurred from December 2021 to June 2022, and this study involved 180 days of follow-up. A total of 320 healthy individuals, aged 18–59 years old, who had been vaccinated at least 6 months previously with only the primary vaccine regimen consisting of two doses of inactivated vaccine and had no history of severe acute respiratory syndrome coronavirus 2 (SARS-CoV-2) infection, were randomly assigned to four equal groups (A–D). Participants in group A, receiving a homologous regimen, were boosted with the same manufacturer as their previous immunization. Participants in group B, also a homologous regimen, were boosted with a different manufacturer than their previous immunization. Participants in group C, following a heterologous regimen, were boosted with an adenovirus type-5-vectored COVID-19 vaccine, while participants in group D, also following a heterologous regimen, were boosted with a recombinant protein-based vaccine. Following that, approximately 5 mL of venous blood was collected from participants at five time points: days 0 (before, vaccination), 14, 28, 90, and 180 after booster immunization (Figure 1). The study was approved by the Fujian Provincial Center for Disease Control and Prevention [Approval No: Fujian Provincial Center for Disease Control and Prevention Ethical Review Approval (2021) No.(021)].

### Vaccines

The inactivated COVID-19 vaccines in this cohort included Sinopharm BIBP (BIBP) and CoronaVac (COVac) were produced by Changchun Institute of Biological Products (Changchun, China, batch number 202108 J0511) and Sinovac Life Sciences Co., Ltd. (Beijing, China, batch number A202109055), respectively. The Convidecia adenovirus type-5-vectored COVID-19 vaccine (Convidecia) was produced by CanSino Biologics Inc. (Tianjin, China, batch number NCOV202109054V). The recombinant protein-based vaccine used was Zifivax recombinant novel coronavirus vaccine (CHO cells) (Zifivax), produced by Anhui Zhifei Longcom Biopharmaceutical Co., Ltd. (Anhui, China, batch number B202109224).

### Procedure

Blood samples collected on site were placed into non-anticoagulant tubes before serum separation. Approximately 5 mL of venous blood was collected each time and left to stand at room temperature, for 2 h. The samples were centrifuged at 3000 rpm, for 5 min. The supernatant was drawn with a sterile pipette and divided into two aliquots, one of which was placed in serum tube A and the other in tube B. Tube A contained at least 0.5 mL of the serum, and the remaining portion was preserved in tube B. The two tubes were cryopreserved at −20°C in separate serum boxes. All serum samples in tube A were uniformly transported to the Biosafety Level 3 (BSL-3) laboratory at Fujian Provincial Center for Disease Control and Prevention for testing. The serum samples in tube B were temporarily cryopreserved at Zhangping City Center for Disease Control and Prevention.

The levels of neutralizing antibodies against live SARS-CoV-2 were assessed by measuring the reduction in the cytopathic effect (CPE) in Vero E6 cells infected with infectious SARS-CoV-2 strain. The serum samples were inactivated at 56°C for 30 min and then successively diluted from 1:2 to 1:1024 in duplicate, with a final volume of 50 μL in a 96-well microplate. An equal volume of 100 TCID_50_ virus was added to each well for neutralizing in a 37°C incubator for 2 h. Then, the neutralizing solution was pipetted to a new well with a monolayer of Vero E6 cells in a 96-well plate and incubated for 5 days at 37°C. The neutralizing antibody was evaluated with the NT_50_ (50% neutralization titer, the reciprocal of the highest dilution protecting 50% of the cells from virus challenge) by calculating the CPE on cells. Positivity for antibodies was defined by the NT_50_ titer≥1:4. The reference serum provided by the China National Institutes for Food and Drug Control served as a positive control and was tested at each instance as an internal quality control measure. All the samples were tested with wild-type SARS-CoV-2 (WT). Serum samples from days 14 and 180 were collected from at least 30 individuals in each group and tested against variant strains, including the Delta variant AY.23 (Delta) and the Omicron variant BA.5.2 (Omicron).

### Statistical analyses

We analyzed the secondary outcomes of immunogenicity at three periods: during days 14–28 (period 1), days 29–90 (period 2), and days 91–180 (period 3). The geometric mean titers (GMTs) with a 95% confidence interval (CI) of the neutralizing antibody were estimated at days 14, 28, 90, and 180 in the four groups. Natural logarithmic transformation of antibody titer was performed. Normal distribution of the data was tested using the Kolmogorov–Smirnov test. For non-normal distributed data, the Kruskal–Wallis test and Friedman test were used. Hypothesis testing was two-sided, and *p*-values of less than 0.05 were significant. Statistical analyses were conducted using SPSS (version 20.0) and GraphPad Prism 8.0.1.

## Results

### Study participants

A total of 320 healthy subjects, aged 18 to 59 years, were recruited for the study and randomly divided into four groups. The baseline characteristics of the participants were similar across the four groups (14). Of these, 310 individuals completed 180 days of follow-up, with 74 participants in group A, 79 participants in group B, 78 participants in group C, and 79 participants in group D.

### Dynamics of neutralizing antibody responses against wild-type SARS-CoV-2

**Figure 2:**
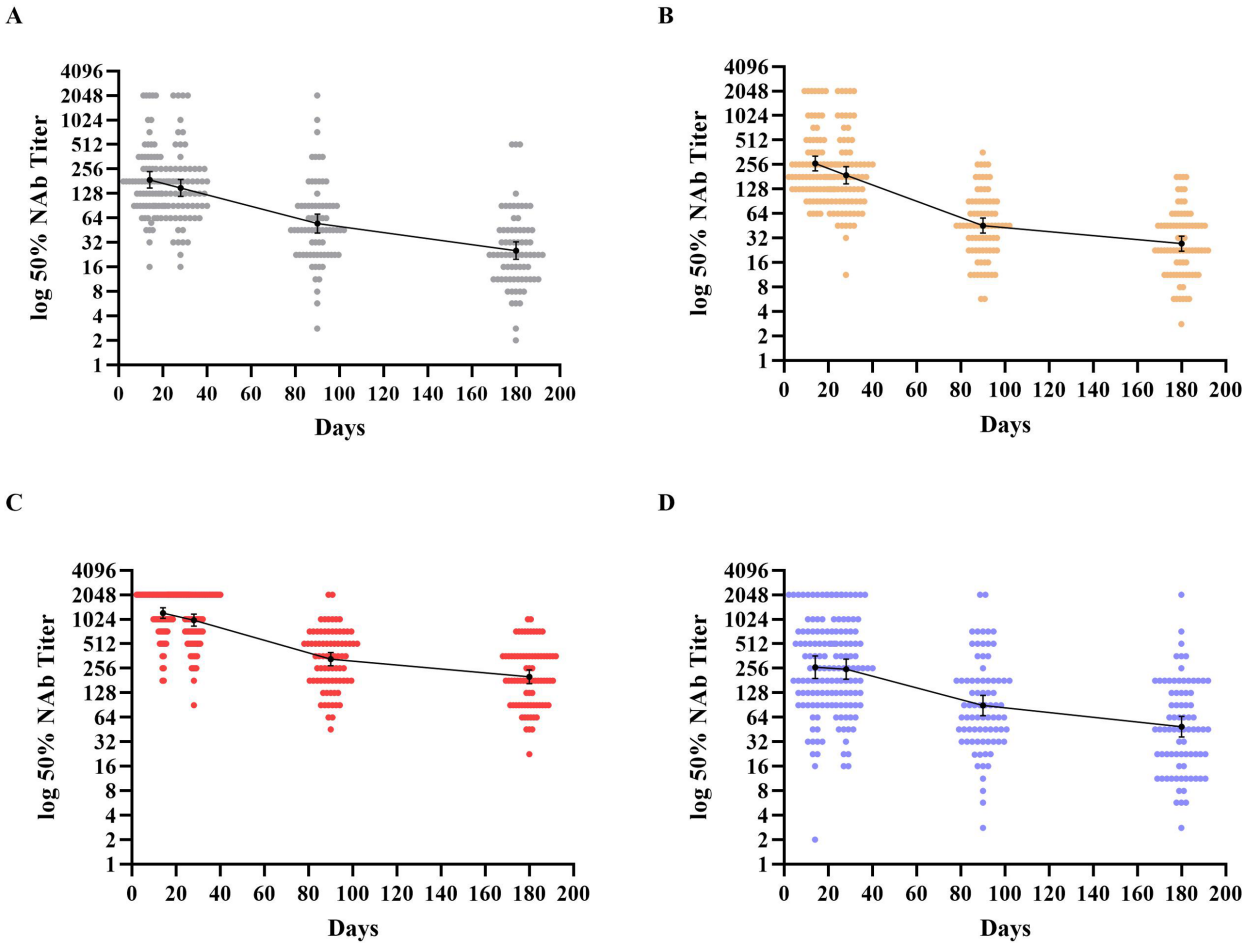
Distribution of neutralizing antibodies 180 days after receipt of the third dose of the booster vaccine. **(A–D)** Show the geometric mean titers (GMTs) of neutralizing antibodies against the wild-type SARS-CoV-2 throughout the study period across the four groups. Participants in groups A and B were boosted with the inactivated vaccine (BIBP/COVac), participants in group C were boosted with an adenovirus vector vaccine (Convidecia), and participants in group D were boosted with the recombinant protein-based vaccine (Zifivax). Dots represent individual observed serum samples. I bars indicate 95% confidence intervals.

To explore the dynamic changes in neutralizing antibody responses of homologous and heterologous boosters to inactivated COVID-19 vaccine, we evaluated the neutralizing antibody titers development at different time points. The highest titers after receipt of the booster dose were all observed on day 14, so this was defined as the peak period. A substantial reduction in the GMTs of neutralizing antibody titers at each time in all groups was observed, which culminated in decreases by factors of 7.41 in group A with a GMT of 25.43 (95%CI, 19.71 to 32.25), 9.66 in group B with a GMT of 27.33 (95%CI, 22.29 to 33.34), 6.07 in group C with a GMT of 201.38 (95%CI, 166.18 to 240.46), and 5.39 in group D with a GMT of 48.97 (95%CI, 36.54 to 65.02), after 6 months. Participants in group C, who were boosted with Convidecia, exhibited higher neutralizing antibody levels than individuals boosted with the inactivated vaccine and Zifivax at each time point of the study (see Figure 2 and Supplementary Tables S1, S2).

The neutralizing antibody kinetics across all groups showed that the GMTs of neutralizing antibodies substantially reduced within 90 days post-boost, followed by a slower decline thereafter. The decrease values from days 14 to 90 composed most proportion in 6 months of follow-up, with proportions of 82.12, 92.34, 87.29, and 81.24% in groups A to D, respectively. However, the decay rates of GMT in each group varied across the three periods. In groups A–C, the decay rates every 2 weeks from the peak to the end of study period 1 were 20.35, 28.03, and 18.12%, respectively. In period 2, the decline slowed, with decay rates of 15.91, 19.01, and 16.73% every 2 weeks, respectively. In period 3, the decay further slowed, with rates of 8.89, 6.64, and 6.53% every 2 weeks, respectively. In group D, the neutralization experienced a slight decline of 5.54% every 2 weeks in period 1, which then shifted to a significant reduction of 16.04% every 2 weeks in period 2, followed by a slower rate of decline at 7.40% every 2 weeks for the subsequent period.

### Neutralizing antibody responses against SARS-CoV-2 variants

To assess the persistent capacity of neutralizing antibodies against the Delta and Omicron variants, individuals from each group were randomly selected for testing their neutralizing antibodies against the variants at the peak period (day 14) and at the end of the study period (day 180). The number of individuals in group A to D was 31, 32, 36, and 39, respectively.

**Figure 3:**
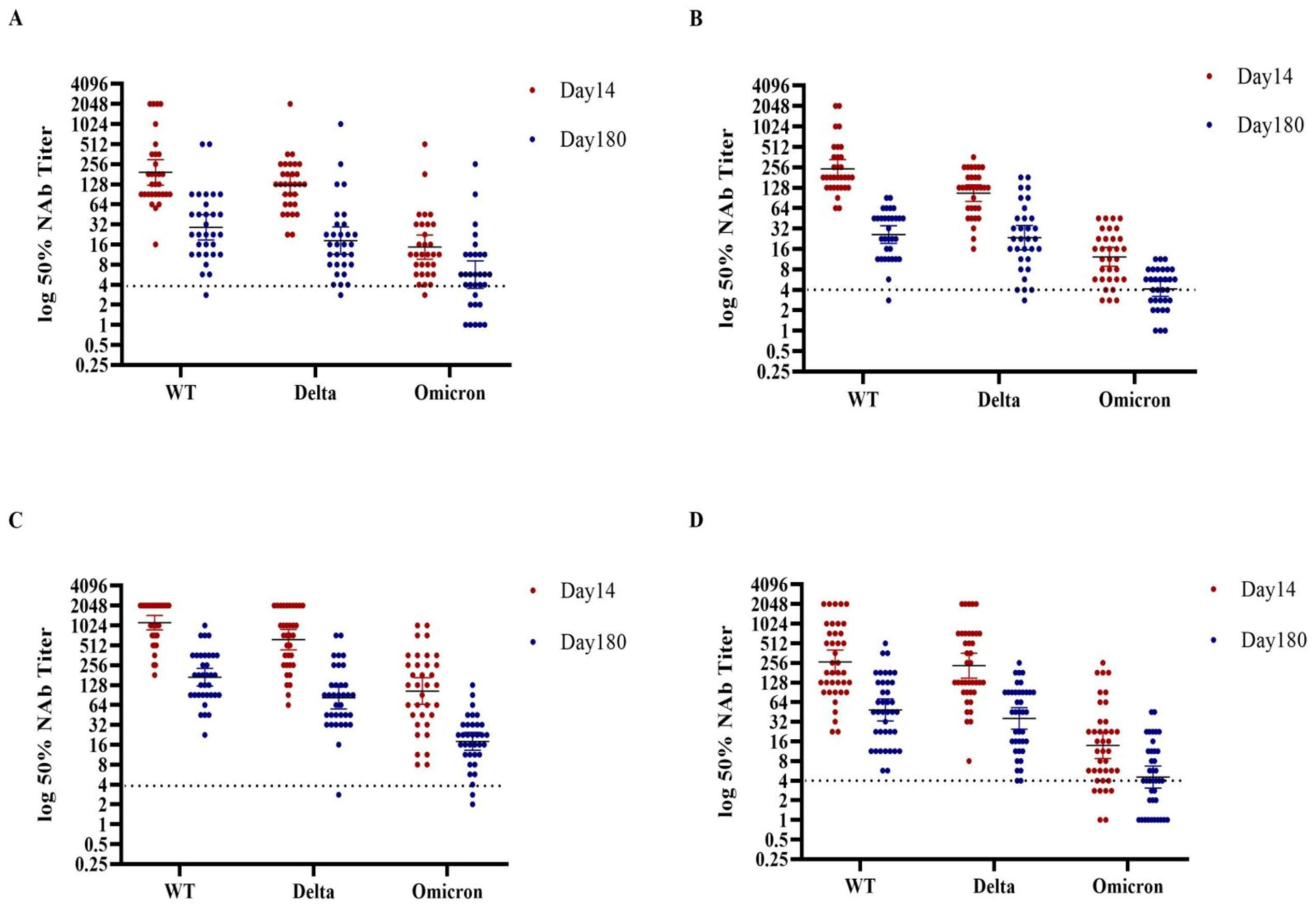
Distribution of neutralizing antibodies against three kinds of SARS-CoV-2 at days 14 and 180 after receipt of a third dose of the booster vaccine in the four groups. **(A–D)** Show the GMTs of neutralizing antibodies against three kinds of SARS-CoV-2 at days 14 and 180 after receipt of the third dose of the booster vaccine in the four groups. Participants in groups A and B were boosted with the inactivated vaccine (BIBP/COVac), participants in group C were boosted with the adenovirus vector vaccine (Convidecia), and participants in group D were boosted with the recombinant protein-based vaccine (Zifivax). Dots represent individual observed serum samples. I bars indicate 95% confidence intervals. The Kruskal–Wallis test and Friedman test were utilized to statistically analyze the differences in GMTs among the various groups.

After 14 days post-boost, the neutralizing antibody titers against the Delta variant in groups A and D were slightly lower compared to those observed for the WT neutralizing antibody titers, while, groups B and C exhibited significantly reduced titers. In contrast, the Omicron variant provoked a much more pronounced decrease by factors of 13.17, 19.88, 10.78, and 19.12 in neutralizing antibody levels across four groups respectively, when compared to their WT levels (see Figure 3 and Supplementary Tables S3, S4). When exploring the impact of different immunization strategies, the results revealed numerically higher GMTs of neutralizing antibodies against both Delta and Omicron variants following heterologous immunization with Convidecia as a booster in group C. These GMTs were compared to those achieved through homologous boost immunization with the inactivated vaccines in groups A and B. Specifically, for Delta variants, the GMT in group C was 629.15 (95%CI, 440.1–882.52), which was notably higher than the 124.14 (95%CI, 91.35–167.1) in group A and 111.43 (95%CI, 85.07–141.52) in group B. Similarly, for Omicron variants, the GMT in group C was 108.13 (95%CI, 68.97–163.14), significantly surpassing the 14.71 (95%CI, 10.17–21.12) in group A and 11.92 (95%CI, 8.88–15.83) in group B. However, when considering heterologous immunization with Zifivax in Group D, no statistically significant differences were observed in comparison to the homologous boost immunization regimen. In group D, the GMT for Delta variants was 232.16 (95%CI, 153.64–356.59), while for Omicron variants was 13.87 (95%CI, 9.02–21.75) (see Supplementary Table S5).

At the end of the study period, the decrease in neutralizing antibodies against both WT and Delta variants was over 80% in all four groups, compared to the participants in the same group at 14 days of post-boost. Participants who were administered the Convidecia booster exhibited higher levels of neutralizing antibodies compared to those who received booster with other regimens. The GMT of neutralizing antibodies against the WT and Delta variants was 169.22 (95%CI,130.34–233.66) and 81.4 (95%CI,56.34–121.52), respectively (see Supplementary Table S4). From days 14 to 180, the decay rates of neutralizing antibodies against the three kinds of the SARS-CoV-2 viruses showed that participants with a higher peak level tended to have a higher decrease. The decrease in neutralizing antibodies against Omicron was 82.97% among participants with the Convidecia booster, compared to 63.98 ~ 67.41% in those boosted with other regimens. The GMT of neutralizing antibodies against Omicron was 18.3 (95%CI,13.86–25.09), higher than that of the other regimens. The Convidecia booster in group C provided higher and more durable neutralizing antibody responses against SARS-CoV-2 variants.

## Discussion

In this report, we compared the 180-day duration of neutralizing antibodies to live SARS-CoV-2 following homologous and heterologous third COVID-19 vaccine dose schedules in populations who received the inactivated COVID-19 vaccine as their initial two doses. The neutralizing antibody titers of individuals who received booster immunization followed a general trend: antibodies peaked at 14 days after the boost, declined drastically within the first 90 days, then slowed down in the subsequent 90 days. The decay rate varied across different time intervals. Regardless of the type of boost immunization, the ability to neutralize SARS-CoV-2 variants was significantly lower than against the WT strain. We found that heterologous immunization with Convidecia could maintain much higher levels of neutralizing antibodies against the three kinds of SARS-CoV-2 variants than homologous immunization, although it may be associated with a higher decay rate. Based on these data, a heterologous prime-boost vaccination with Convidecia after priming with an inactivated COVID-19 vaccine could potentially offer stronger and more durable protection against SARS-CoV-2 as compared to a third dose of inactivated vaccine.

The neutralizing antibody level of live SARS-CoV-2 is considered to positively correlate with protection from the COVID-19 vaccine in real-world vaccinations and can assist in predicting immune protection (15, 16). Multiple studies have shown that regardless of the type of COVID-19 vaccine used in the primary immunization, neutralizing antibodies against SARS-CoV-2 wane out over time, indicating a decrease in vaccine efficacy. The estimated half-life of live virus-neutralizing antibody for mRNA-1273 after two doses was 66 days (17). After receipt of two doses of the BNT162b2 vaccine, the neutralizing antibody level decreased rapidly by 3 months (18). Although, the Ad26.COV2.S vaccine elicited durable humoral immune responses, the neutralizing antibody decreased at 8 months after immunization (19). Our findings like previous studies (20, 21) demonstrated that the neutralizing antibody titer decreased to an extremely low level after prime immunization of inactivated COVID-19 vaccines by 6 months, which greatly reduced their protective effect. The booster immunization indeed induced a strong immune response with a significant increase in the neutralizing antibody titer in a short time, we found that no matter what booster vaccine was used in the individuals, their antibodies had a substantial increase, and the heterologous boosting elicited more robust immune responses than the homologous boosting did, which were consistent with other findings (22–25). According to our 180 days of follow-up, the kinetics data of neutralizing antibody titers against live SARS-CoV-2 showed that the antibodies began to decrease at 28 days after boosting, declined drastically by 90 days, and then slowed down during the next 90 days. This suggests that the third dose of COVID-19 vaccines did not prevent the attenuation of antibodies, and humoral responses are expected to decay over time. Therefore, long-term vaccine protection calls for immunization schedules that elicit a high peak level of antibodies in a short time, with a slow descent. Our study suggested that the heterologous boost with the viral vector vaccine Convidecia as the third dose booster could elicit and maintain higher neutralizing antibodies during the follow-up than the inactivated vaccine and Zifivax boosters. The finding was consistent with previous cohort studies, which reported higher anti-spike IgG and neutralizing antibody response following heterologous Ad26 booster compared with BNT in participants who received BNT/BNT (26, 27). These studies also reported a higher CD8^+^ T-cell response and a slower humoral decay after Ad26 than BNT.

Since the emergence of SARS-CoV-2, a number of variants have continually emerged as the virus evolves and adapts to the human population (28). Mutations in the spike protein can lead to structural and physiochemical changes that, in turn, affect the binding affinity and interaction with ACE2, as well as the efficacy of neutralizing antibodies (29–31). In comparison to the WT SARS-CoV-2, the Delta variant has 8 mutations of the S protein, including 2 within the RBD, while the Omicron variant has 30 spike mutations, including 16 within the RBD (32). The studies have demonstrated that various single mutations of the Omicron variant can impair neutralizing antibodies of different epitope groups, allowing Omicron to evade considerable humoral immunity levels (33, 34). Several studies have reported the reduction of the neutralizing capacity against Delta and Omicron variants were observed in individuals after initial immunization with BBIBP-CorV, CoronaVac, BNT162b2, mRNA-1273, and ChAdOx1-S (35–38). It is encouraging that the third booster has been demonstrated to elicit more neutralizing antibodies, helping to reduce the escape of Delta and Omicron variants and improving protection, particularly with the heterologous booster (39–42). Our results revealed that 14 days post-boost immunization, the capability of neutralizing antibodies induced by boosters against the Delta and Omicron variants decreased compared to against the WT strain, especially for the Omicron variant, which showed a steep reduction in neutralizing capacity. After 180 days of follow-up, the heterologous Convidecia booster maintained higher neutralizing antibodies against both the Delta and Omicron variants than the homologous and Zifivax boosters, which was consistent with the results against the WT strain. Our results are similar to those from previous studies (26, 43), indicating that heterologous boosters of adenovirus-vectored vaccines would further improve Omicron cross-neutralization by eliciting higher antibodies. Although the vaccine effectiveness for the Omicron variant was notably lower than for other variants (44), the booster vaccines could provide neutralizing antibody stable protection against hospitalizations and mortality induced by the Omicron variant (45).

However, there were some limitations to this study. First, the subjects were between 18 and 59 years old, which did not encompass all age groups. These individuals may differ from older populations according to characteristics that could confound our estimates of the vaccine’s immune response. Second, this study only focused on the dynamics of neutralizing antibodies. It would be valuable to test total IgG and IgG subsets to determine skewed immune responses for each of these platforms and to gather further data on memory and cellular responses. Third, vaccine efficacy was not assessed, as information on COVID-19 morbidity and mortality following the booster vaccination was not available in this study due to the limited number of cases in China. Fourth, we detected neutralization activity against the wild-type, Delta and Omicron, however, due to a shortage of live virus samples, we do not have data on the latest variants.

In the post-pandemic period, the peak antibody titers and long-term protection may become a higher priority in choosing which vaccines to use in booster programs. In this study, we found that the neutralizing antibodies decayed by approximately 80% from peak level to 180 days of follow-up. The heterologous boost with the viral vector Convidecia, following two doses of inactivated COVID-19 vaccine, preserved higher antibody titers than the inactivated vaccines or recombinant novel coronavirus vaccine boosters throughout the study. The neutralizing capacity against the Delta and Omicron variants following Convidecia was stronger than that of BIBP, COVac, and Zifivax. This suggests that national immunization committees might consider heterologous adenovirus vector vaccines targeting SARS-CoV-2 variants or other infections as a strategy to boost individuals and maintain higher antibody levels for a longer period.

## Data Availability

The original contributions presented in the study are included in the article/Supplementary material, further inquiries can be directed to the corresponding authors.

## References

[1] World Health Organization. WHO Coronavirus (COVID-19) Dashboard. Available at: http://www.who.int/emergencies/diseases/novel-coronavirus-2019 (Accessed January 14, 2024)

[2] Fan Q Nie Z Xie S. Reflections on the new types of COVID-19 vaccines in the face of variants, breakthrough infection, and vaccine hesitancy. Med J Wuhan Univ. (2023) 44:1429–34. doi: 10.14188/j.1671-8852.2022.0858

[3] Al-Sheboul SA Brown B Shboul Y Fricke I Imarogbe C Alzoubi KH. An immunological review of SARS-CoV-2 infection and vaccine serology: innate and adaptive responses to mRNA, adenovirus, inactivated and protein subunit vaccines. Vaccine. (2022) 11:51. doi: 10.3390/vaccines11010051, PMID: 36679897 PMC9865970

[4] Zhang Y Zeng G Pan H Li C Yaling H Chu K. Safety, tolerability, and immunogenicity of an inactivated SARS-CoV-2 vaccine in healthy adults aged 18–59 years: a randomised, double-blind, placebo-controlled, phase 1/2 clinical trial. Lancet Infect Dis. (2021) 21:181–92. doi: 10.1016/S1473-3099(20)30843-4, PMID: 33217362 PMC7832443

[5] Xia S Zhang Y Wang Y Wang H Yang Y Gao GF. Safety and immunogenicity of an inactivated SARS-CoV-2 vaccine, BBIBP-CorV: a randomised, double-blind, placebo-controlled, phase 1/2 trial. Lancet Infect Dis. (2020) 21:39–51. doi: 10.1016/S1473-3099(20)30831-8, PMID: 33069281 PMC7561304

[6] Li Z Xiang T Liang B Deng H Wang H Feng X. Characterization of SARS-CoV-2-specific humoral and cellular immune responses induced by inactivated COVID-19 vaccines in a real-world setting. Front Immunol. (2021) 12:802858. doi: 10.3389/fimmu.2021.802858, PMID: 35003131 PMC8727357

[7] Al Kaabi N Oulhaj A Ganesan S Al Hosani FI Najim O Ibrahim H. Effectiveness of BBIBP-CorV vaccine against severe outcomes of COVID-19 in Abu Dhabi, United Arab Emirates. Nat Commun. (2022) 13:3215. doi: 10.1038/s41467-022-30835-1, PMID: 35680857 PMC9184465

[8] Collier A-RY Jingyou Y McMahan K Liu J Chandrashekar A Maron JS. Differential kinetics of immune responses elicited by Covid-19 vaccines. N Engl J Med. (2021) 385:2010–2. doi: 10.1056/NEJMc2115596, PMID: 34648703 PMC8531985

[9] Pegu A Conceptualization, Formal analysis, Investigation, Methodology, Project administration O’Connell SE Schmidt SD O’Dell S Talana CA. Durability of mRNA-1273 vaccine induced antibodies against SARS-CoV-2 variants. Science. (2021) 373:1372–7. doi: 10.1126/science.abj4176, PMID: 34385356 PMC8691522

[10] Cheng ZJ Huang H Zheng P Xue M Ma J Zhan Z. Humoral immune response of BBIBP COVID-19 vaccination before and after the booster immunization. Allergy. (2022) 77:2404–14. doi: 10.1111/all.15271, PMID: 35255171 PMC9111230

[11] Peng Q Zhou R Wang Y Zhao M Liu N Li S. Waning immune responses against SARS-CoV-2 variants of concern among vaccinees in Hong Kong. EBioMedicine. (2022) 77:103904. doi: 10.1016/j.ebiom.2022.103904, PMID: 35248996 PMC8893246

[12] Tartof SY Slezak JM Fischer H Hong V Ackerson BK Ranasinghe ON. Effectiveness of mRNA BNT162b2 COVID-19 vaccine up to 6 months in a large integrated health system in the USA: a retrospective cohort study. Lancet. (2021) 398:1407–16. doi: 10.1016/S0140-6736(21)02183-8, PMID: 34619098 PMC8489881

[13] Cohn BA Cirillo PM Murphy CC Krigbaum NY Wallace AW. SARS-CoV-2 vaccine protection and deaths among US veterans during 2021. Science. (2022) 375:331–6. doi: 10.1126/science.abm0620, PMID: 34735261 PMC9836205

[14] Xie FQ Li JR Yang XH Chen ZF Zhang HR Huang RD. Assessment of the immunogenicity of COVID-19 heterogeneous booster vaccination following the full immunization of inactivated vaccines. J Biol Regul Homeost Agents. (2023) 37:2377–84. doi: 10.23812/j.biol.regul.homeost.agents.20233705.234

[15] Khoury DS Cromer D Reynaldi A Schlub TE Wheatley AK Juno JA. Neutralizing antibody levels are highly predictive of immune protection from symptomatic SARS-CoV-2 infection. Nat Med. (2021) 27:1205–11. doi: 10.1038/s41591-021-01377-8, PMID: 34002089

[16] Gilbert PB Montefiori DC McDermott AB Fong Y Benkeser D Deng W. Immune correlates analysis of the mRNA-1273 COVID-19 vaccine efficacy clinical trial. Science. (2022) 375:43–50. doi: 10.1126/science.abm3425, PMID: 34812653 PMC9017870

[17] Rose ND Suthar MS Makowski M Connell SO McDermott AB Flach B. Antibody persistence through 6 months after the second dose of mRNA-1273 vaccine for Covid-19. N Engl J Med. (2021) 384:2259–61. doi: 10.1056/NEJMc2103916, PMID: 33822494 PMC8524784

[18] Levin EG Lustig Y Cohen C Fluss R Indenbaum V Amit S. Waning immune humoral response to BNT162b2 Covid-19 vaccine over 6 months. N Engl J Med. (2021) 385:e84. doi: 10.1056/NEJMoa2114583, PMID: 34614326 PMC8522797

[19] Barouch DH Stephenson KE Sadoff J Yu J Chang A Gebre M. Durable humoral and cellular immune responses 8 months after Ad26.COV2.S vaccination. N Engl J Med. (2021) 385:951–3. doi: 10.1056/NEJMc2108829, PMID: 34260834 PMC8314733

[20] Zeng G Qianhui W Pan H Li M Yang J Wang L. Immunogenicity and safety of a third dose of CoronaVac, and immune persistence of a two-dose schedule, in healthy adults: interim results from two single-Centre, double-blind, randomised, placebo-controlled phase 2 clinical trials. Lancet Infect Dis. (2022) 22:483–95. doi: 10.1016/S1473-3099(21)00681-2, PMID: 34890537 PMC8651254

[21] Wen GP Zhu M Li LR Li X-J Ye HM Zhou Y-L. Homologous booster immunization with an inactivated vaccine induced robust antibody response in healthcare workers: a retrospective study. Front Immunol. (2023) 14:1099629. doi: 10.3389/fimmu.2023.1099629. eCollection 2023, PMID: 36817474 PMC9935570

[22] Zhang Y Yang Y Qiao N Wang X Ding L Zhu X. Early assessment of the safety and immunogenicity of a third dose (booster) of COVID-19 immunization in Chinese adults. Front Med. (2022) 16:93–101. doi: 10.1007/s11684-021-0914-x, PMID: 35122211 PMC8815383

[23] Ai J Zhang Y Zhang H Zhang Q Zhangfan F Lin K. Safety and immunogenicity of a third-dose homologous BBIBP-CorV boosting vaccination: interim results from a prospective open-label study. Emerg Microbes Infect. (2022) 11:639–47. doi: 10.1080/22221751.2022.2025746, PMID: 35034582 PMC8881062

[24] Atmar RL Lyke KE Deming ME Jackson LA Branche AR El Sahly HM. Homologous and heterologous Covid-19 booster vaccinations. N Engl J Med. (2022) 386:1046–57. doi: 10.1056/NEJMoa2116414, PMID: 35081293 PMC8820244

[25] Clemens SAC Weckx L Clemens R Mendes AVA Souza AR Silveira MBV. Heterologous versus homologous COVID-19 booster vaccination in previous recipients of two doses of CoronaVac COVID-19 vaccine in Brazil (RHH-001): a phase 4, non-inferiority, single blind, randomised study. Lancet. (2022) 399:521–9. doi: 10.1016/S0140-6736(22)00094-0, PMID: 35074136 PMC8782575

[26] Sabrina Tan C Collier A-RY Jingyou Y Liu J Chandrashekar A McMahan K. Durability of heterologous and homologous COVID-19 vaccine boosts. JAMA Netw Open. (2022) 5:e2226335. doi: 10.1001/jamanetworkopen.2022.26335, PMID: 35947380 PMC9366542

[27] Liu X Munro APS Wright A Feng S Janani L Aley PK. Persistence of immune responses after heterologous and homologous third COVID-19 vaccine dose schedules in the UK: eight-month analyses of the COV-BOOST trial. *Clinical*. Trial. (2023) 87:18–26. doi: 10.1016/j.jinf.2023.04.012, PMID: 37085049 PMC10116128

[28] Singh J Pandit P McArthur AG Banerjee A Mossman K. Evolutionary trajectory of SARS-CoV-2 and emerging variants. Virol J. (2021) 18:166. doi: 10.1186/s12985-021-01633-w, PMID: 34389034 PMC8361246

[29] Harvey WT Carabelli AM Jackson B Gupta RK Thomson EC Harrison EM. SARS-CoV-2 variants, spike mutations and immune escape. Nat Rev Microbiol. (2021) 19:409–24. doi: 10.1038/s41579-021-00573-0, PMID: 34075212 PMC8167834

[30] Ding C He J Zhang X Jiang C Sun Y Zhang Y. Crucial mutations of spike protein on SARS-CoV-2 evolved to variant strains escaping neutralization of convalescent plasmas and RBD-specific monoclonal antibodies. Front Immunol. (2021) 12:693775. doi: 10.3389/fimmu.2021.693775, PMID: 34484190 PMC8416052

[31] Pitsillou E Liang JJ Beh RC Hung A Karagiannis TC. Molecular dynamics simulations highlight the altered binding landscape at the spike-ACE2 interface between the Delta and omicron variants compared to the SARS-CoV-2 original strain. Comput Biol Med. (2022) 149:106035. doi: 10.1016/j.compbiomed.2022.106035, PMID: 36055162 PMC9420038

[32] Kumar S Thambiraja TS Karuppanan K Subramaniam G. Omicron and Delta variant of SARS-CoV-2: a comparative computational study of spike protein. J Med Virol. (2022) 94:1641–9. doi: 10.1002/jmv.27526, PMID: 34914115

[33] Planas D Saunders N Maes P Guivel-Benhassine F Planchais C Buchrieser J. Considerable escape of SARS-CoV-2 omicron to antibody neutralization. Nature. (2022) 602:671–5. doi: 10.1038/s41586-021-04389-z, PMID: 35016199

[34] Cao Y Wang J Jian F Xiao T Song W Yisimayi A. Omicron escapes the majority of existing SARS-CoV-2 neutralizing antibodies. Nature. (2022) 602:657–63. doi: 10.1038/s41586-021-04385-3, PMID: 35016194 PMC8866119

[35] Abdool Karim SS de Oliveira T. New SARS-CoV-2 variants - clinical, public health, and vaccine implications. N Engl J Med. (2021) 384:1866–8. doi: 10.1056/NEJMc2100362, PMID: 33761203 PMC8008749

[36] Cele S Jackson L Khoury DS Khan K Moyo-Gwete T Tegally H. Omicron extensively but incompletely escapes Pfizer BNT162b2 neutralization. Nature. (2022) 602:654–6. doi: 10.1038/s41586-021-04387-1, PMID: 35016196 PMC8866126

[37] Ai J Zhang H Zhang Y Lin K Zhang Y Wu J. Omicron variant showed lower neutralizing sensitivity than other SARS-CoV-2 variants to immune sera elicited by vaccines after boost. Emerg Microbes Infect. (2022) 11:337–43. doi: 10.1080/22221751.2021.2022440, PMID: 34935594 PMC8788341

[38] Chen Y Chen L Yin S Tao Y Zhu L Tong X. The third dose of CoronVac vaccination induces broad and potent adaptive immune responses that recognize SARS-CoV-2 Delta and omicron variants. Emerg Microbes Infect. (2022) 11:1524–36. doi: 10.1080/22221751.2022.2081614, PMID: 35608053 PMC9176682

[39] Wang X Zhao X Song J Wu J Zhu Y Li M. Homologous or heterologous booster of inactivated vaccine reduces SARS-CoV-2 omicron variant escape from neutralizing antibodies. Emerg Microbes Infect. (2022) 11:477–81. doi: 10.1080/22221751.2022.2030200, PMID: 35034583 PMC8820826

[40] Schultz BM Melo-González F Duarte LF Gálvez NMS Pacheco GA Soto JA. A booster dose of CoronaVac increases neutralizing antibodies and T cells that Recognize Delta and omicron variants of concern. MBio. (2022) 13:e0142322. doi: 10.1128/mbio.01423-22 35946814 PMC9426482

[41] Abu-Raddad LJ Chemaitelly H Ayoub HH AlMukdad S Yassine HM Al-Khatib HA. Effect of mRNA vaccine boosters against SARS-CoV-2 omicron infection in Qatar. N Engl J Med. (2022) 386:1804–16. doi: 10.1056/NEJMoa2200797, PMID: 35263534 PMC8929389

[42] Andrews N Stowe J Kirsebom F Toffa S Rickeard T Gallagher E. Covid-19 vaccine effectiveness against the omicron (B.1.1.529) variant. N Engl J Med. (2022) 386:1532–46. doi: 10.1056/NEJMoa2119451, PMID: 35249272 PMC8908811

[43] Le Gars M Hendriks J Sadoff J Ryser M Struyf F Douoguih M. Immunogenicity and efficacy of Ad26.COV2.S: An adenoviral vector-based COVID-19 vaccine. Immunol Rev. (2022) 310:47–60. doi: 10.1111/imr.13088, PMID: 35689434 PMC9349621

[44] Wu N Joyal-Desmarais K Ribeiro PAB Vieira AM Stojanovic J Sanuade C. Long-term effectiveness of COVID-19 vaccines against infections, hospitalisations, and mortality in adults: findings from a rapid living systematic evidence synthesis and meta-analysis up to December, 2022. Lancet Respir Med. (2023) 11:439–52. doi: 10.1016/S2213-2600(23)00015-2, PMID: 36780914 PMC9917454

[45] Zhou R Liu N Li X Peng Q Yiu CK Huang H. Three-dose vaccination-induced immune responses protect against SARS-CoV-2 omicron BA.2: a population-based study in Hong Kong. Lancet Reg Health West Pac. (2023) 32:100660. doi: 10.1016/j.lanwpc.2022.100660, PMID: 36591327 PMC9786166

