# Supplementary material for: Waning neutralizing antibodies through 180 days after homologous and heterologous boosters of inactivated COVID-19 vaccine
Source: Front Public Health. 2025 Jan 28;13:1478627. doi: 10.3389/fpubh.2025.1478627 (PMC11811089; doi:10.3389/fpubh.2025.1478627)
Supplement: Supplementary file 1 [file Table_1.DOCX]

######

###### **Table1 Neutralizing Antibody titers and positive rates in different groups at different time points.**

| **Time** | **Characteristics** | **Group A** | **Group B** | **Group C** | **Group D** |
| --- | --- | --- | --- | --- | --- |
| Day 14 | GMT (95%CI) | 188.46 (148.37-239.41) | 263.97 (217.08-324.34) | 1223.16 (1048.77-1394.03) | 263.97 (190.05-357.09) |
|  | NO. Positive rate (%) | 74 (100.0) | 79 (100.0) | 78 (100.0) | 78 (98.7) |
| Day 28 | GMT (95%CI) | 150.09 (116.73-191.44) | 189.97 (150.45-241.32) | 1001.46 (841.79-1179.12) | 249.34 (190.2-334.4) |
|  | NO. Positive rate (%) | 74 (100.0) | 79 (100.0) | 78(100.0) | 79 (100.0) |
| Day 90 | GMT (95%CI) | 54.57 (41.8-70.91) | 45.45 (36.82-55.55) | 331.24 (272.65-398.68) | 89.29 (67.74-120.05) |
|  | NO. Positive rate (%) | 73 (98.6) | 79 (100.0) | 78(100.0) | 78 (98.7) |
| Day 180 | GMT (95%CI) | 25.43 (19.71-32.25) | 27.33 (22.29-33.34) | 201.38 (166.18-240.46) | 48.97 (36.54-65.02) |
|  | NO. Positive rate (%) | 72 (97.3) | 78 (98.7) | 78(100.0) | 78 (98.7) |

**Table2 Comparison of logarithmic values of GMTs of WT NAb titers across the groups at different time points.**

| Time | Pairwise comparison | H | P |  | Time | Pairwise comparison | H | P |
| --- | --- | --- | --- | --- | --- | --- | --- | --- |
| Day 14 | Group B&A | -1.684 | 0.553 |  | Day 90 | Group B&A | 0.815 | 1.000 |
|  | Group C&A | -9.377 | ＜0.001 |  |  | Group C&A | -8.645 | ＜0.001 |
|  | GroupD&A | -2.099 | 0.215 |  |  | GroupD&A | -2.412 | 0.095 |
|  | Group C&B | -7.826 | ＜0.001 |  |  | Group C&B | -9.615 | ＜0.001 |
|  | Group D&B | -0.422 | 1.000 |  |  | Group D&B | -3.281 | 0.006 |
|  | Group D&C | 7.405 | ＜0.001 |  |  | Group D&C | 6.344 | ＜0.001 |
| Day 28 | Group B&A | -1.196 | 1.000 |  | Day 180 | Group B&A | -0.430 | 1.000 |
|  | Group C&A | -9.294 | ＜0.001 |  |  | Group C&A | -9.640 | ＜0.001 |
|  | GroupD&A | -2.709 | 0.041 |  |  | GroupD&A | -3.152 | 0.010 |
|  | Group C&B | -8.236 | ＜0.001 |  |  | Group C&B | -9.365 | ＜0.001 |
|  | Group D&B | -1.538 | 0.744 |  |  | Group D&B | -2.768 | 0.034 |
|  | Group D&C | 6.703 | ＜0.001 |  |  | Group D&C | 6.606 | ＜0.001 |

######

###### **Table3 NAb titers against different SARS-CoV-2 strains and positive rates in different groups at different time points.**

| **Strains** | **Time** | **Characteristics** | **Group A** | **Group B** | **Group C** | **Group D** |
| --- | --- | --- | --- | --- | --- | --- |
| Wild type | Day 14 | GMT (95%CI) | 192.73 (127.14 -292.42) | 242.49 (182.55 -331.95) | 1105.97 (861.07 -1404.47) | 265.24 (175.25 -388.01) |
|  |  | NO. Positive rate (%) | 31 (100.0) | 32 (100.0) | 36 (100.0) | 39 (100.0) |
|  | Day 180 | GMT (95%CI) | 28.93 (18.53 -44.45) | 26.04 (19.71 -34.36) | 169.22 (130.34 -233.66) | 48.59 (34.06 -69.81) |
|  |  | NO. Positive rate (%) | 30 (96.8) | 31 (96.9) | 36 (100.0) | 39 (100.0) |
|  |  | Decay rate | 87.46 | 88.43 | 84.30 | 81.68 |
| Delta variant | Day 14 | GMT (95%CI) | 123.78 (87.52 -174.18) | 106.48 (80.38 -138.86) | 608.86 (433.34 -870.38) | 232.16 (151.7 -354.89) |
|  |  | NO. Positive rate (%) | 31 (100.0) | 32 (100.0) | 36 (100.0) | 39 (100.0) |
|  | Day 180 | GMT (95%CI) | 18.29 (11.82 -29.6) | 23.36 (16.14 -35.73) | 81.4 (56.34 -121.52) | 35.92 (25.16 -52.12) |
|  |  | NO. Positive rate (%) | 30(96.8) | 31 (96.9) | 35 (97.2) | 39 (100.0) |
|  |  | Decay rate | 81.09 | 83.79 | 86.66 | 84.53 |
| Omicron variant | Day 14 | GMT (95%CI) | 14.63 (9.96 -22.32) | 12.2 (8.78 -16.63) | 102.56 (67.02 -160.16) | 13.87 (9.26 -22.29) |
|  |  | NO. Positive rate (%) | 30 (96.8) | 29 (90.6) | 36 (100.0) | 33 (84.6) |
|  | Day 180 | GMT (95%CI) | 5.66 (3.6 -9.09) | 4.13 (3.18 -5.22) | 18.3 (13.86 -25.09) | 4.52 (3.13 -6.63) |
|  |  | NO. Positive rate (%) | 22 (71.0) | 20 (62.5) | 34 (94.4) | 24 (61.5) |
|  |  | Decay rate | 63.98 | 64.26 | 82.97 | 67.41 |

**Table4 Comparison of logarithmic values of GMTs of NAb titers against the different strains in the groups at different times.**

| Day14 | | | | | | | | |
| --- | --- | --- | --- | --- | --- | --- | --- | --- |
| Group | Pairwise comparison | H | P |  | Group | Pairwise comparison | H | P |
| A | WT&Delta | 1.397 | 0.487 |  | C | WT&Delta | 2.416 | 0.047 |
|  | WT&Omicron | 6.604 | ＜0.001 |  |  | WT&Omicron | 7.484 | ＜0.001 |
|  | Delta&Omicron | 5.207 | ＜0.001 |  |  | Delta&Omicron | 5.068 | ＜0.001 |
| B | WT&Delta | 2.625 | 0.026 |  | D | WT&Delta | 0.793 | 1.000 |
|  | WT&Omicron | 7.312 | ＜0.001 |  |  | WT&Omicron | 6.850 | ＜0.001 |
|  | Delta&Omicron | 4.688 | ＜0.001 |  |  | Delta&Omicron | 6.058 | ＜0.001 |
| Day180 | | | | | | | | |
| Group | Pairwise comparison | H | P |  | Group | Pairwise comparison | H | P |
| A | WT&Delta | 1.905 | 0.170 |  | C | WT&Delta | 3,182 | 0.004 |
|  | WT&Omicron | 6.287 | ＜0.001 |  |  | WT&Omicron | 7.601 | ＜0.001 |
|  | Delta&Omicron | 4.382 | ＜0.001 |  |  | Delta&Omicron | 4.419 | ＜0.001 |
| B | WT&Delta | 0.250 | 1.000 |  | D | WT&Delta | 2.151 | 0.094 |
|  | WT&Omicron | 5.938 | ＜0.001 |  |  | WT&Omicron | 7.699 | ＜0.001 |
|  | Delta&Omicron | 5.688 | ＜0.001 |  |  | Delta&Omicron | 5.548 | ＜0.001 |

**Table5 Comparison of logarithmic values of GMTs of NAb titers against different strains across the groups at different times.**

| Strains | Time | Pairwise comparison | H | P |  | Time | Pairwise comparison | H | P |
| --- | --- | --- | --- | --- | --- | --- | --- | --- | --- |
| Delta variant | Day 14 | Group B&A | 0.233 | 1.000 |  | Day 180 | Group B&A | -1.584 | 0.679 |
|  |  | Group C&A | -5.484 | ＜0.001 |  |  | Group C&A | -5.358 | ＜0.001 |
|  |  | GroupD&A | -2.064 | 0.234 |  |  | GroupD&A | -2.727 | 0.038 |
|  |  | Group C&B | -5.763 | ＜0.001 |  |  | Group C&B | -3.781 | 0.001 |
|  |  | Group D&B | -2.320 | 0.122 |  |  | Group D&B | -1.110 | 1.000 |
|  |  | Group D&C | 3.567 | 0.002 |  |  | Group D&C | 2.759 | 0.035 |
| Omicron variant | Day 14 | Group B&A | -0.622 | 1.000 |  | Day 180 | Group B&A | 1.134 | 1.000 |
|  |  | Group C&A | -5.190 | ＜0.001 |  |  | Group C&A | -4.391 | ＜0.001 |
|  |  | GroupD&A | -0.159 | 1.000 |  |  | GroupD&A | -0.579 | 1.000 |
|  |  | Group C&B | -5.697 | ＜0.001 |  |  | Group C&B | -5.557 | ＜0.001 |
|  |  | Group D&B | -0.354 | 1.000 |  |  | Group D&B | -0.602 | 1.000 |
|  |  | Group D&C | 5.579 | ＜0.001 |  |  | Group D&C | 5.178 | ＜0.001 |
